# Supplementary material for: Severe hyperbilirubinemia is associated with higher risk of contrast-related acute kidney injury following contrast-enhanced computed tomography
Source: PLoS One. 2020 Apr 15;15(4):e0231264. doi: 10.1371/journal.pone.0231264 (PMC7159198; doi:10.1371/journal.pone.0231264)
Supplement: S1 Fig — (AUC = 0.579, with 34.09% of sensitivity and 78.75% of specificity). (DOC) [file pone.0231264.s001.doc]

Figure 1. ROC curve for total bilirubin>1.2 mg/dl to predict acute kidney injury after contrast-enhanced computed tomography. (AUC=0.579, with 34.09% of sensitivity and 78.75% of specificity)
