# Supplementary material for: Severe hyperbilirubinemia is associated with higher risk of contrast-related acute kidney injury following contrast-enhanced computed tomography
Source: PLoS One. 2020 Apr 15;15(4):e0231264. doi: 10.1371/journal.pone.0231264 (PMC7159198; doi:10.1371/journal.pone.0231264)

Figure 2. All patients (n=9496) and patients with serum bilirubin > 2 mg/dl (n=1368) divided by different liver conditions.

2A. All patients


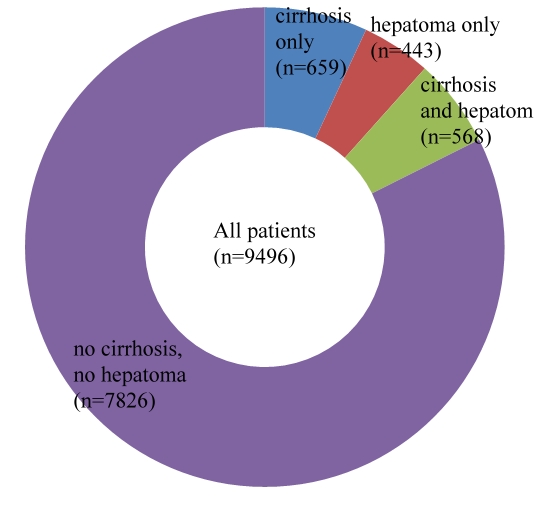


2B. Patients with total bilirubin> 2mg/dl


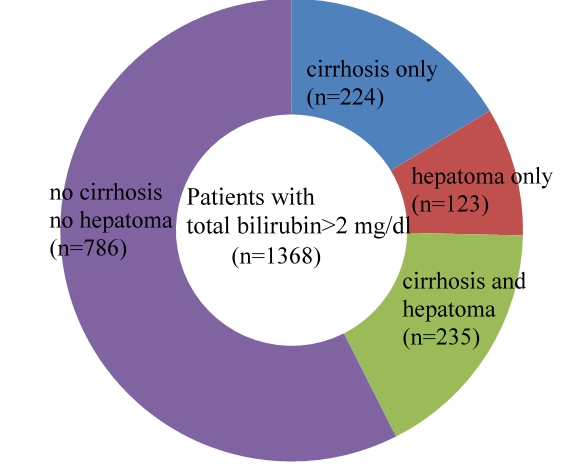

Supplement: S2 Fig — (DOC) [file pone.0231264.s002.doc]
