# Supplementary material for: Severe hyperbilirubinemia is associated with higher risk of contrast-related acute kidney injury following contrast-enhanced computed tomography
Source: PLoS One. 2020 Apr 15;15(4):e0231264. doi: 10.1371/journal.pone.0231264 (PMC7159198; doi:10.1371/journal.pone.0231264)
Supplement: S1 Table — (DOC) [file pone.0231264.s003.doc]

Table 1. AKI and urgent dialysis within 30 days after contrast-enhanced computed tomography in patients with cirrhosis or hepatoma.

|  | | | | | | | | | |
| --- | --- | --- | --- | --- | --- | --- | --- | --- | --- |
|  | Bilt≤1.2  (n=560) | | 1.2<Bilt≤2  (n=208) | | Bilt>2  (n=459) | | Total  (n=1227) | | *P* value |
| **Patients with cirrhosis** (N=1227) |  |  |  |  |  |  |  |  |  |
| AKI | 37 | (6.6%) | 15 | (7.2%) | 94 | (20.5%) | 146 | (11.9%) | **<0.001**** |
| dialysis within 30 days | 30 | (5.4%) | 13 | (6.3%) | 37 | (8.1%) | 80 | (6.5%) | 0.217 |
| **Patient with hepatoma (N=1011)** | | | | | | | | | |
| AKI | 32 | (6.4%) | 14 | (9.2%) | 75 | (20.9%) | 121 | (12.0%) | **<0.001**** |
| dialysis within 30 days | 20 | (4.0%) | 7 | (4.6%) | 20 | (5.6%) | 47 | (4.6%) | 0.549 |
