# Supplementary material for: Severe hyperbilirubinemia is associated with higher risk of contrast-related acute kidney injury following contrast-enhanced computed tomography
Source: PLoS One. 2020 Apr 15;15(4):e0231264. doi: 10.1371/journal.pone.0231264 (PMC7159198; doi:10.1371/journal.pone.0231264)
Supplement: S2 Table — (DOC) [file pone.0231264.s004.doc]

Table 2. Baseline characteristics of patients with total bilirubin> 2mg /dl (n=1368) divided to liver conditions

|  | Cirrhosis only (n=224) | | Liver cancer only  (n=123) | | Both cirrhosis and  liver cancer (n=235) | | No cirrhosis and  no liver cancer (n=786) | | Total  (n=1368) | | *P* value |
| --- | --- | --- | --- | --- | --- | --- | --- | --- | --- | --- | --- |
| **Age (years)** | 59.32 | ±15.39 | 69.15 | ±12.78 | 64.67 | ±12.52 | 67.59 | ±16.81 | 65.88 | ±15.89 | **<0.001**** |
| **≥65 years** | 80 | (35.7%) | 79 | (64.2%) | 121 | (51.5%) | 485 | (61.7%) | 765 | (55.9%) | **<0.001**** |
| **Female** | 64 | (28.6%) | 41 | (33.3%) | 57 | (24.3%) | 253 | (32.2%) | 415 | (30.3%) | 0.100 |
| **Stages of CKD** |  |  |  |  |  |  |  |  |  |  | 0.186 |
| 1 | 95 | (42.4%) | 60 | (48.8%) | 99 | (42.1%) | 287 | (36.5%) | 541 | (39.5%) |  |
| 2 | 54 | (24.1%) | 35 | (28.5%) | 60 | (25.5%) | 222 | (28.2%) | 371 | (27.1%) |  |
| 3a | 25 | (11.2%) | 8 | (6.5%) | 32 | (13.6%) | 111 | (14.1%) | 176 | (12.9%) |  |
| 3b | 23 | (10.3%) | 8 | (6.5%) | 22 | (9.4%) | 76 | (9.7%) | 129 | (9.4%) |  |
| 4 | 21 | (9.4%) | 10 | (8.1%) | 18 | (7.7%) | 57 | (7.3%) | 106 | (7.7%) |  |
| 5 | 6 | (2.7%) | 2 | (1.6%) | 4 | (1.7%) | 33 | (4.2%) | 45 | (3.3%) |  |
| **Laboratory data of blood** |  |  |  |  |  |  |  |  |  |  |  |
| Hemoglobin (g/dl) | 11.67 | ±2.79 | 11.55 | ±2.39 | 11.53 | ±2.46 | 12.34 | ±2.85 | 12.02 | ±2.76 | **<0.001**** |
| Albumin (g/dl) | 2.88 | ±0.73 | 3.19 | ±0.69 | 2.97 | ±0.59 | 3.25 | ±0.73 | 3.12 | ±0.72 | **<0.001**** |
| Calcium (mg/dl) | 7.50 | ±1.57 | 8.10 | ±1.62 | 7.94 | ±1.52 | 7.94 | ±1.51 | 7.88 | ±1.54 | **0.003**** |
| Sodium (meq/L) | 135.36 | ±6.68 | 134.25 | ±5.42 | 134.26 | ±6.23 | 136.97 | ±5.74 | 136.00 | ±6.07 | **<0.001**** |
| Potassium (mg/dl) | 3.99 | ±0.89 | 4.07 | ±0.71 | 4.17 | ±0.89 | 4.05 | ±0.77 | 4.06 | ±0.81 | 0.099 |
| Uric acid (mg/dl) | 9.33 | ±7.16 | -- |  | 5.51 | ±2.54 | 6.71 | ±2.53 | 6.70 | ±3.14 | 0.230 |
| Prothrombin time (s) | 15.75 | ±5.97 | 12.95 | ±5.19 | 14.90 | ±5.46 | 13.67 | ±8.17 | 14.18 | ±7.20 | **<0.001**** |
| pH | 7.15 | ±0.66 | 6.72 | ±0.79 | 6.91 | ±0.83 | 6.94 | ±0.78 | 6.95 | ±0.78 | **<0.001**** |
| HCO3- (mmo/L) | 22.50 | ±5.29 | 22.96 | ±4.75 | 22.99 | ±4.80 | 22.96 | ±5.03 | 22.87 | ±5.03 | 0.773 |
| **Comorbidity** |  |  |  |  |  |  |  |  |  |  |  |
| Diabetes mellitus | 82 | (36.6%) | 36 | (29.3%) | 79 | (33.6%) | 225 | (28.6%) | 422 | (30.8%) | 0.098 |
| Hypertension | 85 | (37.9%) | 54 | (43.9%) | 72 | (30.6%) | 395 | (50.3%) | 606 | (44.3%) | **<0.001**** |
| Cerebrovascular attack | 19 | (8.5%) | 12 | (9.8%) | 12 | (5.1%) | 89 | (11.3%) | 132 | (9.6%) | **0.038*** |
| Peripheral arterial disease | 4 | (1.8%) | 2 | (1.6%) | 1 | (0.4%) | 21 | (2.7%) | 28 | (2.0%) | 0.187 |
| Colon cancer | 6 | (2.7%) | 10 | (8.1%) | 12 | (5.1%) | 73 | (9.3%) | 101 | (7.4%) | **0.004**** |
| Lung cancer | 11 | (4.9%) | 22 | (17.9%) | 33 | (14.0%) | 73 | (9.3%) | 139 | (10.2%) | **<0.001**** |
| Atrial fibrillation | 19 | (8.5%) | 12 | (9.8%) | 8 | (3.4%) | 91 | (11.6%) | 130 | (9.5%) | **0.002**** |
| Coronary arterial disease | 23 | (10.3%) | 14 | (11.4%) | 24 | (10.2%) | 143 | (18.2%) | 204 | (14.9%) | **0.001**** |
| Myocardial infarction | 5 | (2.2%) | 2 | (1.6%) | 9 | (3.8%) | 42 | (5.3%) | 58 | (4.2%) | 0.080 |
| Shock | 18 | (8.0%) | 5 | (4.1%) | 5 | (2.1%) | 27 | (3.4%) | 55 | (4.0%) | **0.007**** |
| Peritonitis | 21 | (9.4%) | 6 | (4.9%) | 29 | (12.3%) | 16 | (2.0%) | 72 | (5.3%) | **<0.001**** |
| Ascites | 16 | (7.1%) | 4 | (3.3%) | 38 | (16.2%) | 8 | (1.0%) | 66 | (4.8%) | **<0.001**** |
| Gastrointestinal bleeding | 39 | (17.4%) | 10 | (8.1%) | 29 | (12.3%) | 35 | (4.5%) | 113 | (8.3%) | **<0.001**** |
| **Medication** |  |  |  |  |  |  |  |  |  |  |  |
| Non-steroidal anti-inflammatory drugs | 62 | (27.7%) | 51 | (41.5%) | 103 | (43.8%) | 287 | (36.5%) | 503 | (36.8%) | **0.003**** |
| Aspirin | 18 | (8.0%) | 18 | (14.6%) | 17 | (7.2%) | 139 | (17.7%) | 192 | (14.0%) | **<0.001**** |
| Aminoglycoside | 82 | (36.6%) | 77 | (62.6%) | 81 | (34.5%) | 446 | (56.7%) | 686 | (50.1%) | **<0.001**** |
| Loop diuretics | 180 | (80.4%) | 70 | (56.9%) | 207 | (88.1%) | 431 | (54.8%) | 888 | (64.9%) | **<0.001**** |
| Angiotensin-converting-enzyme inhibitor | 21 | (9.4%) | 6 | (4.9%) | 12 | (5.1%) | 81 | (10.3%) | 120 | (8.8%) | **0.034*** |
| Angiotensin receptor blockers | 23 | (10.3%) | 19 | (15.4%) | 22 | (9.4%) | 163 | (20.7%) | 227 | (16.6%) | **<0.001**** |
| Steroid | 31 | (13.8%) | 7 | (5.7%) | 33 | (14.0%) | 108 | (13.7%) | 179 | (13.1%) | 0.089 |
| Statin | 5 | (2.2%) | 6 | (4.9%) | 4 | (1.7%) | 61 | (7.8%) | 76 | (5.6%) | **<0.001**** |
| Ranitidine | 20 | (8.9%) | 10 | (8.1%) | 33 | (14.0%) | 53 | (6.7%) | 116 | (8.5%) | **0.006**** |
| Famotidine | 36 | (16.1%) | 21 | (17.1%) | 55 | (23.4%) | 111 | (14.1%) | 223 | (16.3%) | **0.009**** |
| Fluid replacement > 1000c.c. | 74 | (33.0%) | 17 | (13.8%) | 41 | (17.4%) | 190 | (24.2%) | 322 | (23.5%) | **<0.001**** |
| Acute kidney injury | 45 | (20.1%) | 26 | (21.1%) | 49 | (20.9%) | 105 | (13.4%) | 225 | (16.4%) | **0.005**** |
| Dialysis within 30 days | 28 | (12.5%) | 11 | (8.9%) | 9 | (3.8%) | 50 | (6.4%) | 98 | (7.2%) | **0.002**** |
| Chi-square test. †One-way ANOVA. **P*<0.05, ***P*<0.01 | | | | | | | | | | | |
