# Supplementary material for: Severe hyperbilirubinemia is associated with higher risk of contrast-related acute kidney injury following contrast-enhanced computed tomography
Source: PLoS One. 2020 Apr 15;15(4):e0231264. doi: 10.1371/journal.pone.0231264 (PMC7159198; doi:10.1371/journal.pone.0231264)
Supplement: S3 Table — (DOC) [file pone.0231264.s005.doc]

Table 3 Baseline characteristics of patients without cirrhosis or hepatoma (n=7826) divided to three levels of total bilirubin

|  | | | | | | | | | |
| --- | --- | --- | --- | --- | --- | --- | --- | --- | --- |
|  | Bilt≤1.2  (n=6342) | | 1.2<Bilt≤2  (n=698) | | Bilt>2  (n=786) | | Total  (n=7826) | | *P* value |
| **Age (years)** | 64.19 | ±16.58 | 66.22 | ±17.32 | 67.59 | ±16.81 | 64.71 | ±16.71 | **<0.001**** |
| **≥65 years** | 3289 | (51.9%) | 402 | (57.6%) | 485 | (61.7%) | 4176 | (53.4%) | **<0.001**** |
| **Female** | 2561 | (40.4%) | 229 | (32.8%) | 253 | (32.2%) | 3043 | (38.9%) | **<0.001**** |
| **Stages of CKD** |  |  |  |  |  |  |  |  | **0.010*** |
| 1 | 2451 | (38.6%) | 252 | (36.1%) | 287 | (36.5%) | 2990 | (38.2%) |  |
| 2 | 2035 | (32.1%) | 228 | (32.7%) | 222 | (28.2%) | 2485 | (31.8%) |  |
| 3a | 747 | (11.8%) | 101 | (14.5%) | 111 | (14.1%) | 959 | (12.3%) |  |
| 3b | 552 | (8.7%) | 62 | (8.9%) | 76 | (9.7%) | 690 | (8.8%) |  |
| 4 | 332 | (5.2%) | 41 | (5.9%) | 57 | (7.3%) | 430 | (5.5%) |  |
| 5 | 225 | (3.5%) | 14 | (2.0%) | 33 | (4.2%) | 272 | (3.5%) |  |
| **Laboratory data of blood** |  |  |  |  |  |  |  |  |  |
| Hemoglobin (g/dl) | 12.19 | ±2.53 | 12.85 | ±2.63 | 12.34 | ±2.85 | 12.26 | ±2.58 | **<0.001**** |
| Albumin (g/dl) | 3.49 | ±0.72 | 3.38 | ±0.73 | 3.25 | ±0.73 | 3.45 | ±0.73 | **<0.001**** |
| Calcium (mg/dl) | 8.04 | ±1.63 | 7.86 | ±1.62 | 7.94 | ±1.51 | 8.02 | ±1.62 | **0.022*** |
| Sodium (meq/L) | 138.11 | ±5.58 | 137.45 | ±6.17 | 136.97 | ±5.74 | 137.93 | ±5.66 | **<0.001**** |
| Potassium (mg/dl) | 4.08 | ±0.70 | 3.99 | ±0.75 | 4.05 | ±0.77 | 4.07 | ±0.71 | **0.004**** |
| Uric acid (mg/dl) | 6.54 | ±2.56 | 7.17 | ±3.21 | 6.71 | ±2.53 | 6.59 | ±2.59 | 0.457 |
| Prothrombin time (s) | 11.41 | ±4.73 | 12.48 | ±4.96 | 13.67 | ±8.17 | 11.75 | ±5.28 | **<0.001**** |
| pH | 6.88 | ±0.82 | 6.98 | ±0.78 | 6.94 | ±0.78 | 6.90 | ±0.82 | **0.004**** |
| HCO_3_^-^ (mmo/L) | 23.92 | ±5.27 | 23.64 | ±5.16 | 22.96 | ±5.03 | 23.77 | ±5.23 | **0.001**** |
| **Comorbidity** |  |  |  |  |  |  |  |  |  |
| Diabetes mellitus | 1791 | (28.2%) | 177 | (25.4%) | 225 | (28.6%) | 2193 | (28.0%) | 0.253 |
| Hypertension | 3078 | (48.5%) | 355 | (50.9%) | 395 | (50.3%) | 3828 | (48.9%) | 0.370 |
| Cerebrovascular attack | 994 | (15.7%) | 107 | (15.3%) | 89 | (11.3%) | 1190 | (15.2%) | **0.006**** |
| Peripheral arterial disease | 154 | (2.4%) | 14 | (2.0%) | 21 | (2.7%) | 189 | (2.4%) | 0.697 |
| Colon cancer | 722 | (11.4%) | 56 | (8.0%) | 73 | (9.3%) | 851 | (10.9%) | **0.008**** |
| Lung cancer | 1314 | (20.7%) | 72 | (10.3%) | 73 | (9.3%) | 1459 | (18.6%) | **<0.001**** |
| Atrial fibrillation | 545 | (8.6%) | 105 | (15.0%) | 91 | (11.6%) | 741 | (9.5%) | **<0.001**** |
| Coronary arterial disease | 1020 | (16.1%) | 149 | (21.3%) | 143 | (18.2%) | 1312 | (16.8%) | **0.001**** |
| Myocardial infarction | 311 | (4.9%) | 31 | (4.4%) | 42 | (5.3%) | 384 | (4.9%) | 0.724 |
| Shock | 115 | (1.8%) | 19 | (2.7%) | 27 | (3.4%) | 161 | (2.1%) | **0.004**** |
| Peritonitis | 108 | (1.7%) | 23 | (3.3%) | 16 | (2.0%) | 147 | (1.9%) | **0.012*** |
| Ascites | 34 | (0.5%) | 4 | (0.6%) | 8 | (1.0%) | 46 | (0.6%) | 0.249 |
| Gastrointestinal bleeding | 293 | (4.6%) | 39 | (5.6%) | 35 | (4.5%) | 367 | (4.7%) | 0.490 |
| **Medication** |  |  |  |  |  |  |  |  |  |
| Non-steroidal anti-inflammatory drugs | 3250 | (51.2%) | 274 | (39.3%) | 287 | (36.5%) | 3811 | (48.7%) | **<0.001**** |
| Aspirin | 1255 | (19.8%) | 141 | (20.2%) | 139 | (17.7%) | 1535 | (19.6%) | 0.345 |
| Aminoglycoside | 2737 | (43.2%) | 345 | (49.4%) | 446 | (56.7%) | 3528 | (45.1%) | **<0.001**** |
| Loop diuretics | 3335 | (52.6%) | 392 | (56.2%) | 431 | (54.8%) | 4158 | (53.1%) | 0.120 |
| Angiotensin-converting-enzyme inhibitor | 559 | (8.8%) | 74 | (10.6%) | 81 | (10.3%) | 714 | (9.1%) | 0.143 |
| Angiotensin receptor blockers | 1303 | (20.5%) | 139 | (19.9%) | 163 | (20.7%) | 1605 | (20.5%) | 0.913 |
| Steroid | 1409 | (22.2%) | 125 | (17.9%) | 108 | (13.7%) | 1642 | (21.0%) | **<0.001**** |
| Statin | 637 | (10.0%) | 59 | (8.5%) | 61 | (7.8%) | 757 | (9.7%) | 0.065 |
| Ranitidine | 542 | (8.5%) | 44 | (6.3%) | 53 | (6.7%) | 639 | (8.2%) | **0.037*** |
| Famotidine | 980 | (15.5%) | 96 | (13.8%) | 111 | (14.1%) | 1187 | (15.2%) | 0.341 |
| Fluid replacement > 1000c.c. | 1268 | (20.0%) | 152 | (21.8%) | 190 | (24.2%) | 1610 | (20.6%) | **0.017*** |
| Acute kidney injury | 512 | (8.1%) | 67 | (9.6%) | 105 | (13.4%) | 684 | (8.7%) | **<0.001**** |
| Dialysis within 30 days | 265 | (4.2%) | 32 | (4.6%) | 50 | (6.4%) | 347 | (4.4%) | **0.019*** |

Chi-square test. †One-way ANOVA. **P*<0.05, ***P*<0.01
