# Supplementary material for: Severe hyperbilirubinemia is associated with higher risk of contrast-related acute kidney injury following contrast-enhanced computed tomography
Source: PLoS One. 2020 Apr 15;15(4):e0231264. doi: 10.1371/journal.pone.0231264 (PMC7159198; doi:10.1371/journal.pone.0231264)
Supplement: Attachment — Submitted filename: PONE-D-19-31096R1 comments.docx [file pone.0231264.s007.docx]

*Hepatoma and cirrhosis are strongly associated with high bilirubin levels and AKI. To show*

*that high bilirubin is an independent risk factor, the authors should also show the incidence of*

*CI-AKI as for figure 2A but only in the 10% patients with high serum total bilirubin levels and*

*no hepatoma or cirrhosis compared to <1.2 and 1.3-2 mg/dl groups*

**Thanks for this comment. We re-analyzed this special group (only 10%, without cirrhosis**

**or HCC). We added two more tables in the supplementary file, as table 2 and table 3.**

**Supplementary table 2. Baseline characteristics of patients with total bilirubin> 2mg /dl**

**(n=1368) divided to liver conditions.**

**Supplementary table 3 Baseline characteristics of patients without cirrhosis or hepatoma**

**(n=7826) divided to three levels of total bilirubin**

**In supplementary table 3, the incidences of post-contrast AKI in patients without any**

**liver conditions in three different total bilirubin levels were: 8.1% vs. 9.6% vs. 13.4**

**(p<0.001). We drew this outcome in a new figure 4C to point out the importance of total**

**bilirubin level of all clinical conditions. The result can point out the importance the**

**independent role of total bilirubin >2 on post-contrast AKI.**

**Thanks for your comments.**

**There is a mismatch in the numbers of patients without liver conditions (in the 10% as stated in figure 3). The total number of patients with bilirubin ≥ 2 is n = 1670 page 22 while in the new supplementary table 2 total number of patients with bilirubin ≥ 2 n = 1368. Why is that?**

⚫ *What are the serum levels of conjugated (direct) and unconjugated (indirect) bilirubin? These data can also be informative regarding potential bilirubin conjugation issues leading to*

*CI-AKI.*

**Thanks for this comment. Because this is a retrospective study, we did not regularly**

**collect direct/indirect bilirubin and iron/ferritin (associated with hemoglobin degradation)**

**before contrast-enhanced computed tomography. Indeed, this is a major limitation to our**

**study and we wrote it in the final part of discussion. However, even only with total**

**bilirubin, we can still speculate that there is a merit of this study because of large case**

**numbers and very strong statistical significance after adjusting all comorbidities and**

**laboratory data. In addition, total bilirubin is the most easily obtained data in clinical**

**practice to remind clinicians to avoid contrast related AKI. Moreover, it is a novel**

**finding to inform clinicians that hyperbilirubinemia is an independent risk factor for**

**contrast related AKI in addition to patients with liver dysfunction. This is beyond**

**everyone’s expectation and this point out the importance of this study. We highlight this**

**strong association based on current data. Besides, if no data of bilirubin, we should also**

**check icteric sclera in our daily practice. More detailed role of direct or indirect bilirubin**

**on renal function after contrast exposure will be elucidated in our later study. We added**

**the above explanations in the final part of discussion.**

**I am satisfied with the author's responses. However, measuring unconjugated bilirubin should be highly considered for future studies since changes in conjugated bilirubin might be only the reflection of heme metabolism defect or liver dysfunction which might not be directly linked to AKI.**

Response to Reviewers

⚫ *What are the iron and ferritin levels? Are they associated with CI-AKI?*

**Thanks for your comment. We did not have the data of iron and ferritin because this is a**

**retrospective study. In clinical practice, we did not check iron and ferritin because**

**contrast-enhanced computed tomography. Iron and ferritin levels were associated with**

**hemoglobin degradation, which is suggestive of direct/indirect bilirubin, we will study**

**this part in the later study.**

**I am satisfied with the author's responses.**

⚫ *This manuscript was also difficult to follow since the figures are not numbered, figure legends*

*of 3 and 4 are inverted. There are also many syntax and typos errors.*

**There are still many typos and syntax errors.**

**Sorry for this typo. We exchange figure 3 and figure 4.**

*1. Hepatoma and cirrhosis are strongly associated with high bilirubin levels and AKI. To*

*show that high bilirubin is an independent risk factor, the authors should also show the*

*incidence of CI-AKI as for figure 2A but only in the 10% patients with high serum total*

*bilirubin levels and no hepatoma or cirrhosis compared to <1.2 and 1.3-2 mg/dl groups.*

**Thanks for this comment. We re-analyzed this special group (only 10%, without cirrhosis**

**or HCC). We added two more tables in the supplementary file, as table 2 and table 3.**

**Supplementary table 2. Baseline characteristics of patients with total bilirubin> 2mg /dl**

**(n=1368) divided to liver conditions.**

**Supplementary table 3 Baseline characteristics of patients without cirrhosis or hepatoma**

**(n=7826) divided to three levels of total bilirubin**

**In supplementary table 3, the incidences of post-contrast AKI in patients without any**

**liver conditions in three different total bilirubin levels were: 8.1% vs. 9.6% vs. 13.4**

**(p<0.001). We drew this outcome in a new figure 4C to point out the importance of total**

**bilirubin level of all clinical conditions. The result can point out the importance the**

**independent role of total bilirubin >2 on post-contrast AKI.**

**Thanks for your comments.**

**I am satisfied with the author's responses.**

*2. This study suggests that patients with high serum total bilirubin levels have a higher risk of*

*CI-AKI of which 16.4% of the patients has developed CI-AKI in this group. Can the authors*

*speculate in the discussion on why a large portion of patients in this group did not develop*

*CI-AKI while others did?*

**Because of the incidence of PC-AKI varied according to different baseline conditions.**

**The incidence of PC-AKI in patients with total bilirubin>2.0 mg/dl cannot be considered**

**as too high or too low. The incidence PC-AKI in patients with total bilirubin> 2.0 mg/dl**

**was 16.4%. This is the first study to point out the incidence of PC-AKI regarding**

**hyperbilirubinemia. The incidence of PC-AKI varied from 12 to 50% according to**

**different baseline conditions [2-4, 44-46]. The incidence of PC-AKI in patients with total**

**bilirubin> 2.0 mg/dl maybe relatively low because of the following reasons. First, we**

**recruited both inpatients and outpatients. Overall, this population was all patients**

**receiving CCT with relatively mild disease. Most patients had good baseline renal**

**function (66.6% with baseline eGFR>60 ml/min.1.732m2) and without anemia (12.02 g/dl**

**of mean Hb). Only 30.8% patients had DM and shock was only noticed in 4.0% patients.**

**Second, 33.6% patients had cirrhosis and 26.2% patients had hepatoma. Patients with**

**liver disease may have less skeletal muscle mass, which made less creatine storage,**

**followed by less conversion of creatine to creatinine[47]. Creatinine-based formula may**

**underestimate renal dysfunction[47]. Finally, this is the first study to mention the**

**incidence of PC-AKI in patients with total bilirubin>2.0 mg/dl. Therefore, without**

**previous studies for comparison, the incidence (16.4%) cannot be considered as too high**

**or too low.**

**We added this discussion in the part of discussion. Thanks for your comment.**

**I am satisfied with the author's responses.**

*3. Mechanistically, the major source of bilirubin is from heme after hemoglobin degradation.*

*This bilirubin circulates with serum albumin (unconjugated) while the liver conjugates*

*bilirubin to make it soluble for excretion into the bile. The free bilirubin (unconjugated) is the*

*bilirubin that is contributing the most to the effect associated with bilirubin. As such, one*

*missing piece to better understand the protective or deleterious role of bilirubin is the free vs*

*conjugated bilirubin levels. What are the serum levels of conjugated (direct) and unconjugated*

*(indirect) bilirubin? These data can also be informative regarding potential bilirubin*

*conjugation issues leading to CI-AKI. Along this line, heme is metabolized by heme oxygenase*

*into biliverdin before being converted into bilirubin. These processes release Iron and Carbon*

*monoxide which could reflect also a defect in Heme oxygenase activity. Thus, what are the*

*iron and ferritin levels? Are they associated with CI-AKI?*

**Thanks for this comment. Because this is a retrospective study, we did not regularly**

**collect direct/indirect bilirubin and iron/ferritin (associated with hemoglobin degradation)**

**before contrast-enhanced computed tomography. Indeed, this is a major limitation to our**

**study and we wrote it in the final part of discussion. However, even only with total**

**bilirubin, we can still speculate that there is a merit of this study because of large case**

**numbers and very strong statistical significance after adjusting all comorbidities and**

**laboratory data.**

**In addition, total bilirubin is the most easily obtained data in clinical**

**practice to remind clinicians to avoid contrast related AKI. Moreover, it is a novel**

**finding to inform clinicians that hyperbilirubinemia is an independent risk factor for**

**contrast related AKI in addition to patients with liver dysfunction. This is beyond**

**everyone’s expectation and this point out the importance of this study. Besides, if no data**

**of bilirubin, we should also check icteric sclera in our daily practice. We highlight this**

**strong association based on current data. More detailed role of direct or indirect**

**bilirubin on renal function after contrast exposure will be elucidated in our later study.**

**We added the above explanations in the final part of discussion.**

**This section “However, even only with total bilirubin, we can still speculate that there is a merit of this study because of large case numbers and very strong statistical significance after adjusting all comorbidities and laboratory data.” should be removed from the discussion and the authors should let the readers judge the strength of the manuscript based solely on the data provided.**

**All the sections added in the revised manuscript on pages 17-18 should be re-write for a scientific manuscript discussion not as a response to reviewers.**

**The discussion should be modified accordingly.**

*Minor concerns:*

*In the discussion, the link between PKC and bilirubin and how it is relevant for the present*

*manuscript is not clear.*

**We discussed it because these were studies about the protective function of bilirubin**

**based on protein kinase C (PKC) [Diabetes. 1998;47(6):859-66. American journal of**

**nephrology. 1998;18(4):344-50. Arteriosclerosis, thrombosis, and vascular biology.**

**1997;17(5):969-78]. However, as your comment, the link was weak and these studies were**

**published many years ago. Thus, we deleted this discussion about the PKC.**

**I am satisfied with the author's responses.**

**Other minors comments:**

**The authors should decrease the use of “this is the first study”. It is repetitive and unnecessary.**

Page 18: “Second, the mechanism of direct injury by severe hyperbilirubinemia in CI-AKI remains to be explored. The biological mechanism needs more preclinical studies in the future.” This is redundant…
