# Supplementary material for: Severe hyperbilirubinemia is associated with higher risk of contrast-related acute kidney injury following contrast-enhanced computed tomography
Source: PLoS One. 2020 Apr 15;15(4):e0231264. doi: 10.1371/journal.pone.0231264 (PMC7159198; doi:10.1371/journal.pone.0231264)
Supplement: Attachment — Submitted filename: 20200313 Reply to comments.docx [file pone.0231264.s008.docx]

*1.*There is a mismatch in the numbers of patients without liver conditions (in the 10% as stated in figure 3). The total number of patients with bilirubin ≥ 2 is n = 1670 page 22 while in the new supplementary table 2 total number of patients with bilirubin ≥ 2 n = 1368. Why is that?

Case numbers with different liver conditions were as follows,

- All patients (n=9496): cirrhosis only (n=659), hepatoma only (n=443), both cirrhosis and hepatoma (n=568), and no cirrhosis no hepatoma (n=7826)
- Patients with total bilirubin> 2mg/dl (n=1368): cirrhosis only (n=224), hepatoma only (n=123), both cirrhosis and hepatoma (n=235), and no cirrhosis no hepatoma (n=786)

Therefore, “1670” is cirrhosis only (n=659)+hepatoma only (n=443)+both cirrhosis and hepatoma (n=568) in all patients. We revised the figure legnds of figure 3. Also, we added a supplementary figure 2 (2A for all patients and 2B for patients with total bilirubin>2 mg/dl) .

*2.*There are still many typos and syntax errors.

🡪We revised all text carefully.

*3.* This section “However, even only with total bilirubin, we can still speculate that there is a merit of this study because of large case numbers and very strong statistical significance after adjusting all comorbidities and laboratory data.” should be removed from the discussion and the authors should let the readers judge the strength of the manuscript based solely on the data provided.

🡪According to your suggestion, we deleted it.

4.All the sections added in the revised manuscript on pages 17-18 should be re-write for a scientific manuscript discussion not as a response to reviewers.

The discussion should be modified accordingly.

- We re-wrote and revised this part. Thanks for your comment.

Other minors comments:

The authors should decrease the use of “this is the first study”. It is repetitive and unnecessary.

🡪We decreaed this sentence according to your suggestion.

Page 18: “Second, the mechanism of direct injury by severe hyperbilirubinemia in CI-AKI remains to be explored. The biological mechanism needs more preclinical studies in the future.” This is redundant…

🡪We deleted “The biological mechanism needs more preclinical studies in the future”

Support of this manuscript: This study was funded by grant TCVGH-1063601B and TCVGH-1073604C from Taichung Veterans General Hospital. The funders had no role in study design, data collection and analysis, decision to publish, or preparation of the manuscript
